# Nanoparticle-Based Strategies to Treat Neuro-Inflammation

**DOI:** 10.3390/ma11020270

**Published:** 2018-02-09

**Authors:** Rémy Poupot, Dylan Bergozza, Séverine Fruchon

**Affiliations:** INSERM, U1043, CNRS, U5282, Université de Toulouse, UPS, Centre de Physiopathologie de Toulouse-Purpan, F-31300 Toulouse, France; remy.poupot@inserm.fr (R.P.); dylan.bergozza@univ-tlse3.fr (D.B.)

**Keywords:** nanotechnology, nanoparticles, neuro-inflammation, blood-brain barrier, nanosafety

## Abstract

Neuro-inflammation is a pivotal physio-pathological feature of brain disorders, including neurodegenerative diseases. As such, it is a relevant therapeutic target against which drugs have to be proposed. Targeting neuro-inflammation implies crossing the Blood-Brain Barrier (BBB) to reach the Central Nervous System (CNS). Engineered nanoparticles (ENPs) are promising candidates to carry and deliver drugs to the CNS by crossing the BBB. There are several strategies to design ENPs intended for crossing through the BBB. Herein, we first put nanotechnologies back in their historical context and introduce neuro-inflammation and its consequences in terms of public health. In a second part, we explain how ENPs can get access to the brain and review this area by highlighting recent papers in the field. Finally, after pointing out potential guidelines for preclinical studies involving ENPs, we conclude by opening the debate on the questions of nanosafety and toxicity of these ENPs and in particular on ecotoxicity related to regulatory issues and public concerns.

## 1. Introduction

### 1.1. “Nanotechnology”: An Historical Perspective

It is a generally acknowledged fact that the first report mentioning the neologism “nano-technology” was authored by Pr. Norio Taniguchi in 1974 [1]. Therein, “nano-technology” is defined as the “production technology to get the extra high accuracy and ultra-fine dimensions, i.e., the preciseness and fineness of the order of 1 nanometre”. It is added that “nano-technology mainly consists of the processing of separation, consolidation and deformation of materials by one atom or one molecule”. The true launch of nanotechnology occurred in the 1990s. At that time, a seminal lecture was rediscovered and publicised in the field. This is the now famous lecture “Plenty of room at the bottom” given by Pr. Richard P. Feynman (Nobel Prize in Physics in 1965) at a meeting of the American Physical Society at Caltech on the 29 December 1959 [2]. In his lecture, Feynman emphasized “the problem of manipulating and controlling things on a small scale”, stating that “it is a staggeringly small world that is below”. Strikingly, at least for biologists, part of his demonstration relies on the “marvellous biological system” in which “an information is contained in a very tiny fraction of the cell in the form of long-chain DNA molecules in which approximately 50 atoms are used for one bit of information about the cell”. Feynman also noticed that “biology is not simply writing information; it is doing something about it. A biological system can be exceedingly small. Many of the cells are very tiny, but they are very active; they manufacture various substances; they walk around; they wiggle; and they do all kinds of marvellous things—all on a very small scale”. Moreover, upon the fabrication of devices at the atomic scale, he pointed out that a new class of miniaturized instrumentation would be needed to manipulate and measure the properties of these materials. Indeed, such instruments started to be invented and developed in the 1980s. The scanning tunnelling microscopes (1981) and the atomic force microscopes (1986) provided the ability not only to visualize the individual atoms, but also to move them around. At the same period, Pr. K. Eric Drexler, motivated by the dream of nanoscale assemblers which would be able to build a copy of themselves and of other items, has explored this basic statement and proposed pioneering studies dealing with the principles and mechanisms of molecular nanotechnology [3]. In addition, the tremendous development of computers led to precise simulation of the materials at the nanoscale. These advanced theories, tools, and techniques have opened a wide field of investigation and development now referred to as “nanotechnology”. 

There are several definitions of nanotechnology and products thereof, often generated for specific aims. As a general definition, it is accepted that nanotechnology deals with objects of which at least one dimension is in the nanometre scale (1 to 100 nm). In the field of nanomedicine, it has been proposed to extend this range to 1000 nm [4]. Earlier, Whitesides proposed an amazing arrangement, stating that the right size has to be defined in accordance with the targeted application, once again based on the observation of the biochemical structures and biological functional units [5]. For the first time, a scientific field is not defined by the nature of its goals, but by a size range. Indeed, the importance of the nanometre range has to be pointed out because the related nanosystems have particular properties and functions resulting directly from their small structure [6]. Contrary to the properties of macroscopic structures, which are governed by classical physics, characteristics of nanostructures follow a quantum behaviour. Actually, as the behaviour of electrons depends on the modification of the matter at the nanoscale, it is possible to design and built new materials with predictable, controlled, and tuneable properties.

### 1.2. “Nanotechnology” Today: A Buzzword

However, nanotechnology is not the science of anything in the realm of 1 to 100 nm, because this would include for instance deoxyribonucleic acid and protein technologies, which are part of biology and biochemistry. More generally, all that is sub-cellular in life sciences falls within the nanometre scale. Hence, one must distinguish nanotechnology from “nanoscience” in general, and from biology in particular (although biology was a relevant starting point in Feynman’s seminal lecture, as already stated). Nanotechnology should rather be seen as part of nanoscience. Nanotechnology, as any “technology”, encompasses the idea of something related to the ability of human beings to “engineer” and to “make”. This is orthogonal to the Darwinian natural evolution of life on Earth. Nevertheless, the fine and tuneable control at the atomic level leading to a specific property that nanotechnology tends to exhibit is reminiscent to what is observed in nature. Indeed, evolution has developed and optimized nanoscale processes over millions of years, and, of course, is still going on. The natural matters (both organic and inorganic) are structured, down to the finest details, at the level of atoms. Besides, natural materials display well organized hierarchical structures, which induce intriguing properties such as the strength of bones or the strength and elasticity of spider silk [7]. On the contrary, there is obviously a wide interface between nanotechnology and biotechnology, so called “nanobiotechnology”.

“The meaning of nanotechnology (and nanoscience) has been eroded as its use has become more and more popular, transforming it into a buzzword. Indeed, the risk is that nanotechnology becomes its own justification, as if, from an arbitrary principle, everything is nanotechnology and nanotechnology will undoubtedly turn out for the good” was remarked in 2009 by Rolland et al. [8]. Nowadays, nanotechnology and its engineered nanoparticulate products (engineered nanoparticles, ENPs) are used in mass market products (cosmetics, food, textiles), and in pharmaceutical products (diagnostics, vaccines, drugs). More and more reports warn about toxicity of ENPs and other products engineered from nanotechnology. It is noteworthy that this concern is related to their natural occurrence and to their use within mass market products, but not to their use as therapeutic agents in which toxicity issues are always studied. As we focus on specific therapeutic application of ENPs in neuro-inflammation, we will not address the multi-faceted topic of toxicity but we will discuss it in the last section of this review.

### 1.3. About Neuro-Inflammation

**Figure 1:**
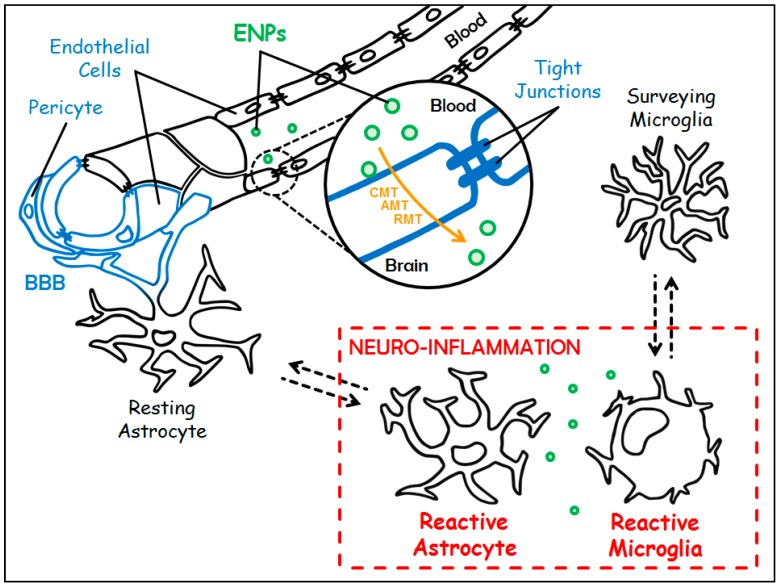
Targeting chronic neuro-inflammation with Engineered Nanoparticles (ENPs). The Blood-Brain Barrier (BBB) in blue is made up of a single layer of endothelial cells (sealed by tight junctions) surrounded by pericytes and astrocytic feet. Under chronic neuro-inflammation, resting astrocytes and surveying microglia switch towards reactive counterparts (in red). Customized ENPs (in green) can cross the BBB either through Carrier-Mediated Transport (CMT), Adsorptive-Mediated Transcytosis (AMT) or Receptor-Mediated Transcytosis (RMT) (in orange) to treat chronic neuro-inflammation.

Inflammation is a physiological process of defense triggered by the body in response to any type of aggression. The brain has long been considered a “privileged immune site” because of both its physical isolation from the periphery by the Blood-Brain Barrier (BBB) and the absence of conventional lymphatic drainage. Nevertheless, the brain is able to mount an inflammatory reaction and, if necessary, an immune response [9]. Several stimuli may initiate the inflammatory response within the Central Nervous System (CNS), so-called neuro-inflammation. These stimuli include Pathogens-Associated Molecular Patterns (PAMPs), and Damage-Associated Molecular Patterns (DAMPs). DAMPs can be physically—or chemically—induced cell damages, or endogenous agents such as modified self-molecules. These PAMPs and DAMPs are recognized by Pattern Recognition Receptors (PPRs), which are expressed by immunocompetent cells within the cerebral parenchyma. These cells are mainly microglial cells and, to a lesser extent, astrocytes [10], and macrophages, the latter being located in the choroid plexus, the meninges, and the perivascular spaces. Usually, under homeostatic condition, microglia are described as “surveying cells” and astrocytes as “resting cells” (Figure 1). Microglia and astrocytes belong to the glial cell family, which also holds oligodendrocytes. Beside their immunological functions, microglia also play a crucial role in both the development and the homeostasis of the CNS [11]. Astrocytes, the most abundant glial cells, fulfill a wide range of functions necessary to trophic support, energy supply, and protection of the neurons. They also participate in the maintenance of the BBB [12]. Following the recognition of danger signals, the immunocompetent cells elicit downstream signaling cascades leading to the activation of pathways that allow the set up of the inflammatory response. Depending on the intensity of the inflammatory response, a process of infiltration of peripheral immune cells (lymphocytes, dendritic cells, and monocytes) can take place. The inflammatory response is a dynamic and organized process which, schematically, takes place in two steps [13]:

(i) The first step is initiated upon recognition of the danger signals by microglia, its purpose is to eliminate the triggering element. It is characterized by the expression of class II antigen presenting molecules (MHC II) and costimulatory molecules (CD80, CD86), and by the secretion of pro-inflammatory cytokines (TNFα, IL-1β, IL-12…), chemokines (CCL2, CCL5…), nitric oxide (NO), and reactive oxygen species (ROS, such as superoxide anions). All are necessary to eradicate the aggressive agent;
(ii) Then comes a phase of resolution of the inflammation characterized by the secretion of anti-inflammatory molecules (among others, the anti-inflammatory cytokines IL-10 and TGFβ), and tissue repair factors. This phase allows the arrest of the acute step, the healing of the injured tissue, and the return to homeostasis. A major difference with the systemic inflammatory response is that this resolution phase mediated by microglia also promotes neuroprotection and neuroreparation. On the one hand, neuroprotection is mediated through the synthesis of neurotrophic factors such as Insulin-like Growth Factor 1 (IGF1), Brain-Derived Neurotrophic Factor (BDNF), and Glial cell-Derived Neurotrophic Factor (GDNF). On the other hand, neuroreparation is mediated through the stimulation of neurogenesis by microglia, and through the plasticity of neural circuits.

As long as it is coordinated, short-lived and finely regulated, this acute neuro-inflammation is beneficial. However, in case of imbalance, for example when there is persistence of the triggering factor or defects in the mechanisms of resolution of the neuro-inflammatory process, the neuro-inflammatory reaction then becomes chronic. At this stage, microglia and astrocytes undergo morphological, phenotypical and functional modifications, and are then named “reactive microglia” and “reactive astrocytes” (Figure 1). The consequences of this chronicity are the sustained release of inflammatory and oxidative mediators, with devastating effects on the cerebral parenchyma and the potential degeneration of the neurons, and the reduction of the production of neurotrophic and neuroprotective factors. Thereby, chronic neuro-inflammation is detrimental and leads to neuronal damage and irreversible tissue injury. 

The scientific community has now recognized that this chronic neuro-inflammation is a central component in almost all neurological disorders, either as a causative factor or as a secondary player [14,15]. In any case, neuro-inflammation actively participates in brain damage by watering the cerebral parenchyma with an arsenal of inflammatory and oxidative molecules that are toxic to neurons. For a long time, neurodegenerative diseases have been considered as cell-autonomous neuronal diseases. However, experimental, epidemiological, and genetic data have clearly demonstrated that chronic neuro-inflammation actively contributes to the cascade of events leading to neuronal degeneration. Therefore, neuro-inflammation is now recognized as a promoter in the etiology and pathogenesis of neurodegenerative diseases such as Alzheimer’s [16] and Parkinson’s diseases [17], and Amyotrophic Lateral Sclerosis [18]. Neuro-inflammation is also involved in the onset of secondary injury after stroke and traumatic brain injury [19,20]. In these pathologies, the damage can be substantial and sustained over time (several months), which leads to neurological impairment.

Neurological disorders affect nearly a billion people worldwide. The latest figures from the World Health Organization (WHO) reveal that stroke is the second leading cause of death in the world and dementia the seventh (Alzheimer’s disease being the main representative of this category) [21]. Importantly, the number of dementia-induced death doubled between 2010 and 2015 (1.54 million deaths worldwide in 2015). This number will continue to increase due to aging of the population and increasing incidence of these pathologies [22]. However, there is still no treatment to cure or even slow down the evolution of the majority of brain disorders (except for epilepsy, depression, schizophrenia, and chronic pain [23]); the ones that exist being simply palliative. Thereby, these disorders represent the largest area of unmet medical need and are a major socio-economic burden. Since neuro-inflammation plays a harmful role in almost all the diseases of the CNS, it represents a relevant therapeutic target [24,25]. Nevertheless, the presence of the BBB limits the access of potential therapies to the brain. Indeed, around 98% of brain-targeting drug candidates do not cross the BBB, and new biotherapy strategies, such as monoclonal antibodies (mAb) and small interfering RNA (siRNA), do not pass over this barrier [26,27]. The BBB is even described by some scientists as the bottleneck of the pharmaceutical development of molecules for the disorders of the CNS [28]. This is one of the reasons which may explain why the drug medical market for illnesses of the CNS is not sufficiently developed [29].

### 1.4. About Blood-Brain Barrier

The brain is located in a non-expandable cavity, and therefore does not support the edema associated with inflammatory reactions. In addition, neurons, which are post-mitotic cells, are extremely sensitive to all disturbances. This is why incoming and outgoing molecules must be extremely controlled. This is the function of the BBB which is undoubtedly the most impermeable barrier of the human body. This physiological boundary separates the cerebral parenchyma from the peripheral blood circulation. It is composed, from the bloodstream to the cerebral parenchyma, of a single layer of endothelial cells surrounded by a thick basal membrane, of pericytes, that stand by the abluminal surface of the endothelial cells, and, finally, of astrocytic feet [30,31] (Figure 1). The main anatomical characteristic of endothelial cells of the BBB is that they are sealed together by tight junctions (Figure 1), therefore forming non-fenestrated capillaries, unlike blood vessels in almost every other part of the body. As a consequence, the passage of molecules between endothelial cells, called paracellular transport, is almost non-existent. As mentioned, the role of this barrier is to protect the brain and it can be considered as a first line of defense. To note, the endothelial cells of the BBB participate in the protection of the brain against the induction of a potentially deleterious neuro-inflammatory response. Indeed, they prevent the diapedesis of peripheral immune cells, since they only weakly express, under homeostatic conditions, leukocyte adhesion molecules [31]. Thus, the function of the BBB is to be an extremely selective filter, limiting macromolecular and cellular traffic and preventing inadvertent access of pathogens, toxins, hormones, and peripheral immune cells. Nevertheless, at the same time, the BBB allows the shipping of nutrients to the cerebral parenchyma and the elimination of waste through active transport systems. Therefore, the BBB is almost a waterproof barrier and only very few molecules, i.e., small lipophilic (<400–500 Da) and hydrophobic compounds (O_2_, CO_2_), get across it. In addition, since paracellular transport is almost non-existent, any passage is made through the endothelial cells and will depend on the physicochemical characteristics of the compound. The other side of the coin is that the passage of therapeutic molecules is also extremely restricted. An additional level of hardship is provided by the presence of P-glycoprotein that belongs to the superfamily of ATP-Binding Cassette (ABC) transporters. The P-glycoprotein is located at the luminal surface of endothelial cells of the BBB. It is an ATP-dependent efflux pump, able to expel in particular exogenous xenobiotic substances out of the endothelial cells [32]. Accordingly, the development of new approaches enabling drug delivery to the brain is a major challenge for our aging societies which have more and more people suffering from neurological diseases [33,34]. 

## 2. Engineered Nanoparticles: Promising Candidates to Tackle Neuro-Inflammation

### 2.1. Different Ways for Engineered Nanoparticles to Access the Central Nervous System

In this context, ENPs, thanks to their particular physicochemical properties, their high chemical stability, their ability to encapsulate a wide variety of both hydrophilic and hydrophobic drugs, and their high functionalization level, are one of the most promising strategies to circumvent the BBB. Indeed, ENPs have opened new avenues and possibilities for drug delivery to the CNS [35]. Reference is made in the literature of “naked” ENPs that are able to spontaneously cross the BBB [36], however, most of them are fine-tuned to allow BBB crossing. Indeed, there are several strategies for ENPs to access the brain parenchyma that can be grouped into the non-invasive methods and the invasive ones. The latter (i.e., opening of the BBB by chemical compound or ultrasound, convection-enhanced delivery, direct inoculation into the brain by either intracerebroventricular or intracerebral injection) will not be discussed here. Indeed, they are difficult to implement in patients, especially in the case of chronic diseases which will require long-term treatment, and they can display potential adverse effects. One of the non-invasive strategies is to harness the endogenous physiological mechanisms of transport of brain-needed molecules across the BBB, which encompass the following pathways [33] (Figure 1):

(i) The Carrier-Mediated Transport (CMT) which is used to carry nutrients or endogenous substances into the brain. To name a few: glucose transporter-1 (GLUT-1/Slc2a1) for the uptake of glucose, and L1 and y^+^ for the uptake of large neutral and cationic essential amino-acids, respectively;
(ii) The Adsorptive-Mediated Transcytosis (AMT) that involves electrostatic interactions between cationic compounds and negative charges of the membrane of endothelial cells prompting the formation of vesicles of endocytosis;
(iii) The Receptor-Mediated Transcytosis (RMT) that relies on the expression of receptors at the luminal plasma membrane of endothelial cells (i.e., directed towards the bloodstream): transferrin receptor (TfR), LDL (Low Density Lipoprotein) Receptor-related Protein 1 and 2 (LRP-1 and -2), insulin receptor and folate receptor. This pathway warrants the entrance of endogenous macromolecules into the CNS.

Another non-invasive strategy is to use a different route of administration, i.e., the intranasal route. Indeed, this non-invasive delivery option allows for the bypass of the BBB and the very fast delivery (in a few minutes) of compounds with pharmacological actions (proteins, oligonucleotides, viral vectors, and ENPs including dendrimers) directly to the CNS. This direct nose-to-brain delivery route is possible thanks to the presence of olfactory and trigeminal nerves, which provide unique connections between the external environment of the nasal cavity and the brain [37,38]. Nevertheless, there are some restraints to the use of this route of administration, including high mucociliary clearance which leads to an abbreviated residence time in the nasal cavity. Moreover, at the nasal level, the low absorption of drugs (which depends on the molecular weight and lipophilicity of the compounds—permeability of hydrophilic drugs being lower), and enzymatic degradation (by the cytochrome P450, proteases, and peptidases) induce a kind of first-pass effect [39]. This is why, even if this route allows direct access to the brain, a delivery system is often necessary, and ENPs can quite fulfill this function. 

The different strategies and criteria set out below should be taken into account when designing ENPs that one wishes to use for brain penetration. In this view, the customization of the ENPs, for example by grafting targeting ligands known to facilitate BBB crossing, seems to be an essential step. Thereby, depending on the degree and type of functionalization they have, ENPs can be assimilated to “Swiss knives”. Indeed, they can gain access to the cerebral parenchyma, target a specific cell population, and deliver their payload for diagnostic and/or therapeutic purposes. 

### 2.2. Engineered Nanoparticles in Action

In the current review, we highlight scientific works that illustrate the breakthrough of ENPs in the field of neuro-inflammation and give recent examples of functionalization which allow ENPs to cross the BBB and to access brain parenchyma. We only deal with scientific studies performed in vivo, omitting those made only in vitro. The different studies we have selected are summarized in Table 1 (ENPs, customizations, cargos, animal models).

Hu et al. have demonstrated that lactoferrin-conjugated (Lf) polyethylene gycol-polylactide-polyglycolide (PEG-PLGA) ENPs (Lf-PEG-PLGA ENPs) have a significantly greater capacity to reach the cerebral parenchyma than unconjugated ENPs [40]. Indeed, PEG is known to improve blood circulation time and Lf is a targeting ligand that promotes receptor-mediated transport through the TfR. Following an intravenous (IV) injection, these Lf-PEG-PLGA ENPs were located in the striatum and the substantia nigra, two affected areas in Parkinson’s disease. Finally, this brain delivery system was used as a carrier for urocortin (a 40 amino acid long peptide related to corticotropin-releasing factor) which has been found to be neuroprotective and to alleviate the symptoms in a rat model of Parkinson’s disease. It should be noted that the authors showed that these ENPs were almost non-toxic for brain cells. The important role of lactoferrin for brain delivery of PEG-PLGA ENPs has also been demonstrated in a more recent study [41].

Tiwari et al. have clearly established that curcumin (Cur) encapsulated in biodegradable PLGA (Cur-PLGA) ENPs is a promising therapeutic approach for Alzheimer’s disease [42]. Curcumin, the main pigment of turmeric (*Curcuma longa*), has interesting anti-inflammatory and anti-oxidant properties for the treatment of inflammatory diseases of the CNS. Nevertheless, its use in therapy is limited because of its poor brain availability, in part due to low BBB permeability. Therefore, the authors have chosen to encapsulate it in highly lipophilic PLGA ENPs to overcome this weakness. Indeed, after intraperitoneal (IP) administration, the brain level of curcumin was higher for the rats injected with Cur-PLGA ENPs compared to those who have received non-encapsulated curcumin. Most notably, in a rat model of Alzheimer’s disease, these Cur-PLGA ENPs have stimulated adult hippocampal neurogenesis and rescued the animal from cognitive decline.

Piperine (PIP), the main active ingredient in pepper, is used in traditional medicine for its anti-inflammatory and anti-microbial properties. The authors used tripolyphosphate cross-linked chitosan (CS) ENPs as a carrier for intranasal delivery of PIP [43]. Indeed, CS is a polysaccharide composed of the random distribution of β-(1-4)-linked d-glucosamine (deacetylated unit) and *N*-acetyl-d-glucosamine (acetylated unit), with particular properties, i.e., biodegradability, biocompatibility, and safety. Moreover, CS is positively-charged which offers both a better interaction with biological membranes and mucoadhesive properties. The PIP-CS ENPs efficacy was evaluated in vivo in a rat model of sporadic dementia of Alzheimer’s type induced by the central injection of colchicine. This study has demonstrated that the nanoformulation was anti-inflammatory (decrease in TNFα production), anti-apoptotic (decreased activity of caspase-3) and antioxidant (increased activity of superoxide dismutase), while this was not the case with the non-formulated PIP. Furthermore, whereas piperine alone is irritant for the nasal mucosa, the PIP-CS ENPs are not muco-irritant. Finally, these PIP-CS ENPs led to an increase in cognitive functions of the animals. 

Hernando et al. have showed that GDNF, which promotes the survival, among others, of dopaminergic neurons (cells that die during the course of Parkinson’s disease) can be addressed to the brain after intranasal administration when it is encapsulated in a nanoformulation composed of nanostructured lipid carrier (NLC) coated with CS and carrying on their surface the cell penetrating peptide TAT (transactivator of transcription) [44]. CS and TAT peptide were chosen to enhance the residence time of the drug in the nasal cavity, to protect it from degradation and to enhance brain delivery. Indeed, only the complete formulation (CS-NLC-TAT-GDNF ENPs) showed therapeutic efficacy in the MPTP mouse model of Parkinson’s disease, i.e., a decreased loss of dopaminergic neurons in the striatum and in the substantia nigra, a decreased number of reactive microglia, and a gain in the motor function. Neither GDNF alone nor the incomplete ENPs (CS-NLC-GDNF and CS-NLC) had these effects, showing the major contribution of TAT peptide for the targeting of GDNF to the brain. 

In a recent study, Kim et al. have exploited the receptor-mediated transcytosis mechanism to send cationic nanoliposomes (scL) containing oligonucleotides or siRNAs to the brain [45]. Instead of using mAb against the TfR as targeting ligand, they grafted single-chain fragment from the variable region of TfR mAb due to smaller size and higher stability (TfRscFv). They first proved in mice, after a single IV injection, that these TfRscFv-scL nanocomplexes, displaying a targeting ligand and containing fluorescent oligonucleotides, can be rapidly addressed to the brain, as early as 6 h. In addition, the fluorescence intensity was significantly higher than the one obtained after the injection of nanocomplexes without targeting moiety. Then, they validated that these liposomes targeting the TfR and containing a siRNA against TNFα had a therapeutic benefit in an acute model of neuro-inflammation induced by the injection of the lipopolysaccharide (LPS). Only 10% of mice pre-treated with non-encapsulated siTNFα survived after LPS administration due to the massive inflammation, whereas the animal mortality was reduced by 90% when the animals were pretreated with nanocomplexes targeting the TfR, compared to those lacking the targeting ligand (in this case, the protective rate was only 50%). 

Tanshinone IIA (TIIA) is the major active component of Chinese medicinal herb Danshen (*Salvia miltiorrhiza*) and has anti-inflammatory properties. Nevertheless, its use for the treatment of neurological disorders including cerebrovascular diseases is curbed by its poor solubility and short plasma half-life. Liu et al. circumvented these hurdles by encapsulating this compound in the biodegradable, FDA-approved polymeric poly lactic acid (PLA) ENPs, which were pegylated and conjugated to cationic bovine serum albumin (CBSA) (CBSA-PEG-TIIA ENPs) [46]. The pharmacokinetic study revealed that ENP-encapsulated TIIA has an increased blood circulation time compared to the non-encapsulated compound, and can be efficiently conveyed to the brain. Based on these early results, the therapeutic efficacy of these CBSA-PEG-TIIA ENPs was then evaluated in a rat model of cerebral ischemia, because it is now widely known that inflammation plays a role in ischemic stroke as an amplifier of the secondary brain injury after cerebral ischemia/reperfusion. This work has clearly demonstrated that CBSA-PEG-TIIA ENPs have significantly abrogated the inflammatory response in this in vivo model: decreased expression of pro-inflammatory cytokines, including TNFα, and of the inflammatory enzyme COX-2, and conversely, increased expression of the anti-inflammatory cytokines IL-10 and TGF-β. Moreover, the animals treated with the nanovehicle showed a decrease in neuronal apoptosis and in infarct size. Thus, the CBSA-PEG-PLA ENPs constitute an effective delivery system for the drug TIIA and they can be used for the treatment of disorders of the CNS including ischemic stroke. 

Although the nasal route allows for the bypass of the BBB and the direct access to the brain, nose-to-brain transport can be impacted by the innate defense mechanism of the airways, namely mucociliary clearance. In order to increase the residence time of ENPs in the nasal cavity, Wen et al. have customized PEG-PLGA ENPs with odorranalectin (OL), which binds to l-fucose expressed on the olfactory epithelium [47]. Using in vivo imaging, they demonstrated that OL-grafted ENPs had a greater ability to localize in the brain following intranasal administration. They used these ENPs to encapsulate the neuropeptide urocortin (see the description of the compound above). They found that the OL-PEG-PLGA-URO ENPs were more efficient in the 6-hydroxydopamine (6-OHDA) model of Parkinson’s disease than the PEG-PLGA-URO ones, the latter being more effective than urocortin alone.

Later on, Yadav et al. demonstrated that, following intranasal injection, the amount of a siRNA against TNFα encapsulated in cationic lipids nanoemulsions (SNE) was significantly higher in the rat brain, and in particular, in the substantia nigra (a brain area involved in Parkinson’s disease) compared to un-encapsulated naked siRNA [48]. They also showed, based on histopathological study, that these cationic nanoemulsions-encapsulated siTNFα are well tolerated by the olfactory and respiratory mucosa of the nasal cavity. Finally, this encapsulation system significantly reduced TNFα and iNOS mRNA expression in a neuro-inflammation model induced by the stereotaxic injection of LPS into the substantia nigra.

Although they do not directly concern neuro-inflammation, it seems important to mention several research works that provide meaningful knowledge on key points for the design and optimization of ENPs in order to overcome the BBB and target the CNS. Voigt et al. have demonstrated in an elegant study using an in vivo confocal neuroimaging technique in living rats, that the most influencing parameter for efficient BBB crossing of Poly-Butyl-CyanoAcrylate (PBCA) polymeric ENPs is the type of surfactant, size and zeta potential having little or no impact [49]. They established that non-ionic tensides (in this study, Tween 80 showed the best efficacy) allow BBB crossing whereas anionic surfactants, such as sodium dodecyl sulfate (SDS), hinder brain uptake. The proposed mechanism put forward by the authors (and by others [50,51,52]) is that this type of non-ionic surfactant interacts with blood circulating apoliproprotein E, which enables them to cross the BBB by lipoprotein receptor-dependent transcytosis (i.e., LRP1), and to enter the brain. Of note, they showed that the ENPs coated with a non-ionic surfactant and also bearing the cationic resin DEAE efficiently crossed the BBB. This occurs probably via adsorptive-mediated transcytosis, in addition to the receptor-mediated mechanism mentioned above. Nevertheless, depending on the type of ENPs, size could be a significant factor. Indeed, it has been shown that the size of gold ENPs coated with insulin (INS-GNPs) influences the brain penetration [53]. In mice, the highest concentration of gold in the brain was gathered 2 h after IV injection of 20 nm gold ENPs (in comparison with 50 and 70 nm ENPs). Finally, we quote an example of a study in which the authors have cleverly combined several approaches in order to optimize and increase the brain uptake of ENPs [54]. They have designed pegylated CS ENPs grafted or not grafted with the mAb OX26 (CS-PEG-OX26 or CS-PEG ENPs) as a targeting ligand. Their idea was:

(i) To increase blood circulating time thanks to PEG;
(ii) To favor adsorptive-mediated transcytosis due to electrostatic interactions between polycationic CS and negatives charges of the membrane of the endothelial cells;
(iii) To allow receptor-mediated transcytosis because of the high selectivity of OX-26 mAb for the highly expressed TfR. Two hours after intraperitoneal (IP) administration, semi-quantitative analysis revealed that ENPs were mostly located in the hippocampus and that the mean of CS-PEG-OX26 ENPs per optical field was two to three times greater than the one of CS-PEG ENPs. The authors made the proof of principle that these ENPs are able to cross the BBB and reach the brain, making them promising drug delivery systems to the CNS.

## 3. Discussion

In light of the literature reviewed herein, several points must be kept in mind when developing and designing ENPs for the treatment of CNS disorders. First, various functionalization of ENPs can be implemented to increase either their ability to cross the BBB [40,45,49,53,54] or their transport from nose-to-brain [43,44]. It is even possible to combine several types of customization to increase the successful targeting of the CNS [44,54]. Secondly, several routes of administration can be envisaged to address ENPs to the brain parenchyma, as it is the case in the examples cited in this review (peritoneal [42,54], intravenous [40,45,46,49,53], or intranasal injection [43,44,47,48]). Conversely, the injection of the nanoparticles directly into the brain (intracerebroventricular injection) should be excluded. Indeed, it will be impossible to conclude whether the nanoparticle has the capacity to reach the brain and, above all, this route of administration will not be easily applicable to humans, especially for chronic CNS disorders that require long-term treatments. At last, the experiments should not only be performed in vitro but should necessarily include in vivo tests. This is why we have chosen to mention only scientific works that respect this last point.

To conclude, based on the reviewed literature and for the results of preclinical studies to be predictive for clinical trials, importance should be given to validate the ability of ENPs to cross the BBB in healthy wild-type animals which therefore have an intact barrier (it should be noted that in all the research articles cited herein, the evaluation of BBB crossing has been carried out in wild-type animals), to choose the animal model the closest to human pathology, and to evaluate the potential neurotoxicity of the ENPs [40,43,48]. Similarly, it is also critical to assess whether ENPs can induce an inflammatory response by themselves (regardless of the therapeutic molecule they carry) [55].

## 4. Conclusions

Nanotechnology generates both attraction and reluctance in the public, related to its technological and economic potentials on the one hand, and its environmental and health incidence on the other hand [56]. Over the years, numerous ENP systems have been approved by the regulatory agencies worldwide, both as therapeutics and diagnostics [57,58]. Nevertheless, it is recognized by the scientific community, and consequently by the regulatory agencies, that the risk assessment rules for non-particulate materials are not relevant, and therefore not sufficient, to evaluate the same material in a nano-particulate form [59,60,61,62]. Exposure of living organisms to nanoparticles has always existed as some nanoparticles have natural origins. Today, ENPs are also incidentally released from industrial and manufacturing processes. Therefore, the level of the different types of ENPs is increasing in our environment, and consequently in our body, with long-term potential deleterious effects which remain unknown. For instance, it has been shown in rats that some ENPs can cross through the placenta when administered to pregnant rats [63]. Therefore, exposure of the mother can have effects on the embryos and then on the offspring. Many reports have shown in different animal models that ENPs can have negative effects on the male germ cells and on the female reproductive system, impairing reproduction and normal embryonic and fetal development (for review, see [64]). Permanently exposed organs can be logically damaged by ENPs. Typically, lung exposure can have adverse respiratory outcomes linked to chronic inflammation, or oxidative stress. Nevertheless, the literature in the field is conflicting and the risk is unclear [65]. Once they have entered the organism, ENPs can be taken up by different types of cells [66] and activate the stress pathway of the endoplasmic reticulum (ER). In turn, ER stress can induce cell apoptosis and development of diseases [67]. ENPs can also display immunotoxicity [68] as the uptake of nanoparticles by different populations of immune cells (especially cells of the innate immunity) can induce sustained and inappropriate immune response of the host [69]. Last but not least, ENPs are an emerging class of environment pollutants coming from the human activities. Environmental factors influence the physical, chemical, and biological transformation of ENPs [70], making it difficult to assess accurately and thoroughly the ecotoxicity of ENPs. Several reviews document the interactions between ENPs and plants [71], and between ENPs and freshwater and marine aquatic organisms [72,73].

There is a constantly increasing literature dealing with nanotoxicity, and, obviously, our purpose to end this mini-review is not to comment exhaustively on this literature. We rather want to alert that, beyond the studies that have shown toxicity of ENPs, little is understood regarding the mechanisms underpinning nanotoxicity. This means that a strong involvement of stakeholders is needed (authorities, industrials, and researchers) to go far beyond the current knowledge, and to reassure the public to not hinder the wide but reasoned use of nanotechnology.

## Figures and Tables

**Table 1:** Compilation of the studies selected for the current review.

ENP Type	Customization (Targeting Ligand)	Therapeutics	Route of Administration and Animal Model	Reference
polyethylene gycol–polylactide–polyglycolide (PEG-PLGA)	Lactoferrin (Lf) targeting the Transferrin Receptor (TfR) on endothelial cells	Urocortin (URO)	Intravenous (IV) Rat model of Parkinson’s disease (PD)	[40]
PEG-PLGA	Lactoferrin (Lf) targeting the TfR on endothelial cells	Shikonin (SHK)	IV Healthy rats only	[41]
PLGA	Non applicable (NA)	Curcumin (Cur)	Intraperitoneal (IP) Rat model of Alzheimer’s disease (AD)	[42]
Tripolyphosphate cross-linked cationic chitosan (CS)	NA	Piperine (PIP)	Intranasal (IN) Rat model of sporadic dementia of AD type	[43]
Nanostructured Lipid Carrier (NLC) coated with cationic CS	TransActivator of Transcription (TAT)	Glial cell-Derived Neurotrophic Factor (GDNF)	IN Mouse model of PD	[44]
cationic nanoliposomes (scL)	Single-chain fragment from the variable region of anti-TfR monoclonal antibody (TfRscFv)	siRNA against TNFα	IV Lipopolysaccharide (LPS)-induced neuro-inflammation in mice	[45]
PEG polymeric poly lactic acid (PLA)	Cationic bovine serum albumin (CBSA)	Tanshinone IIA (TIIA)	Rat model of cerebral ischemic stroke	[46]
PEG-PLGA	Odorranalectin (OL), targeting l-fucose expressed on the olfactory epithelium	URO	IN Rat model of PD	[47]
Cationic lipids nanoemulsions (SNE)	NA	siRNA against TNFα	IN LPS-induced neuro-inflammation in rats	[48]
Poly-Butyl-CyanoAcrylate (PBCA)	Non-ionic surfactants (in particular Tween 80) with or without cationic resin DEAE (both interacting with circulating apoliproprotein E)	NA	IV Healthy rats only	[49]
Gold nanoparticles (GNP)	Insulin (INS)	NA	IV Healthy mice only	[53]
CS-PEG	Anti-TfR monoclonal antibody OX26	NA	IP Healthy mice only	[54]
